# 

**Published:** 1863-07

**Authors:** 


### THE AMERICAN DENTAL CONVENTION,

Will meet at Saratoga, on the 4th of August, when we hope to meet a large number of the profession from both East and West.

We always look forward to these meetings with pleasant anticipations.

Profit and social pleasure, are here always so nicely blended, that those who go can't avoid enjoyment.

The programme of subjects for discussion, is given on another page of this number. They are good, and will doubtless elicit much interesting discussion.

It should be a general outpouring of the profession of the whole country. T.

### AMERICAN DENTAL ASSOCIATION.

This body will meet in Philadelphia, on Tuesday the 28th of this month, and from what we have learned, think there will be a large meeting.

All the local societies will be represented, and by a pretty full representation too

There are many things that will contribute to make this meeting a pleasant and profitable one. All who have ever made the experiment, know that a visit to our brethren of the Quaker City is always accompanied with pleasure.

Almost everybody can go; business has been good, and money abundant, and there is just now, and probably will be through the month of August, somewhat of a lull, so that all can go, without loss in business, and the physical energizing will far more than compensate for the expenditure of money. T.

D. H. PORTER. W. C. PORTER. C. D. PORTER.

### D. H. PORTER & SONS,

MANUFACTURERS OF

### PORCELAIN TEETH,

BRIDGEPORT, CONN.

### PENNSYLVANIA COLLEGE OF DENTAL SURGERY.

### THE EIGHTH ANNUAL SESSION 1863-64.

**Faculty.**

J. D. WHITE, D. D. S.
Emeritus Professor.

T. L. BUCKINGHAM, D. D. S.
Professor of Chemistry and Metallurgy.

C. N. PEIRCE, D. D. S.
Prof. of Dental Physiology & Operative Dentistry.

E. WILDMAN, D. D. S.
Professor of Mechanical Dentistry

G. T. BARKER, D. D. S.
Prof. of Principles of Dental Surgery and Therapeutics,

W. S. FORBES, D. D. S.
Professor of Anatomy aud Physiology.

JAMES TRUMAN, D. D. S.
Demonstrator of Operative Dentistry.

E. N. BAILEY, D. D. S.
Demonstrator of Mechanical Dentistry.

The regular Course will commence on the first Monday of November and continue until the first of March ensuing.

During October the Laboratory will be open, and a Clinical Lecture delivered every Saturday, by one of the Professors, at 3 o'clock, P. M.

The most ample facilities are furnished for a thorough course of practical instruction.

Tickets for the Course, Demonstrators' Tickets included, $100. Matriculation Fee,$5. Diploma Fee, $30.

For further information, address, C. N. PEIRCE, *Dean.*
501 *North Seventh Street, Philadelphia.*

### PHILADELPHIA DENTAL COLLEGE.

### FIRST ANNUAL SESSION, 1863-64.

---

### FACULTY.

C. A. KINGSBURY, D.D.S.,
Professor of Dental Physiology and Operative Dentistry.

THOS. WARDLE, D.D.S.,
Professor of Mechanical Dentistry and Metallurgy.

J. H. McQUILLEN, D.D.S.,
Professor of Anatomy, Physiology and Hygiene.

J. FOSTER FLAGG, D.D.S.,
Professor of Institutes of Dentistry.

HENRY MORTON, A.M,
Professor of Chemistry.

GEO. W. ELLIS, D.D.S.,
Demonstrator of Operative Dentistry.

WM. GORGES, D.D.S.,
Demonstrator of Mechanical Dentistry.

The Laboratory of the College will be open, and preliminary lectures will be delivered by one of the Professors every day during the month of October; the lecture on Wednesday of each week, at 3 o'clock P.M., to be devoted to *Clinical* teaching. The regular Course of instruction will commence on the first Monday of November, and continue until the close of the ensuing February.

The lectures will be amply illustrated by the extensive and *valuable* collections of Anatomical, Pathological, and Mineralogical specimens, and the Philosophical and Chemical apparatus of the incumbents of the various Chairs, and every opportunity will be afforded in the Clinic and Laboratory for obtaining a *practical* knowledge of Operative and Mechanical Dentistry.

### FEES.

| | |
|---|---|
| Matriculation (paid but once) - - - - - | $5 00 |
| Tickets for the Course, including the Demonstrators' - | 100 00 |
| Diploma - - - - - - - - - - | 30 00 |

For further particulars, address J. H. McQUILLEN, DEAN,

July '63 1112 *Arch Street*, *Philadelphia*.

### Ohio College of Dental Surgery.

SESSION OF 1863–4.

Tho regular course of Lectures in this Institution, will commence on the first Monday of November, and close on the 20th of February.

### Faculty.

JAS. TAYLOR, D. D. S. 171 Race Street.
Institutes of Dental Science.

J. TAFT, D. D. S. 172 Race Street
Operative Dentistry.

GEO. EDWIN JONES, M. D. 481 West Seventh Street.
Anatomy and Physiology.

H. R. SMITH, D. D. S. 13 West Fourth Street.
Mechanical Dentistry.

H. A. SMITH, D. D. S. 118 West Sixth Street.
Chemistry and Metallurgy.

E. COLLINS, D. D. S.
Adjunct Prof. of Operative and Mehanical Dentistry.

**Text Books.**—Gray's and Wilson's Anatomy; Carpenter's, Todd and Bowman's Physiology; Williams' Pathology; Fown's, Turner's, Graham's, or Brand and Taylor's Chemistry; Taft's Operative Dentistry; Richardson's Mechanical Dentistry; Harris' Principles and Practice of Dental Science.

### Terms of Admission.

Tickets must be procured at the beginning of the session.

| | |
|---|---|
| Tickets for the course, - - | $100 00 |
| Matriculation Fee, - - | 5 00 |
| Diploma Fee, - - - - | 30 00 |

### Terms of Graduation.

Two courses, (including one of dissections,) with a dental pupilage.
Four years reputable practice will be received as equal to one course.
An acceptable thesis upon some subject on Dental science.
Must possess a good moral character, and be twenty-one years old.

For further information, address J. TAYLOR, *Dean.*
or J. TAFT, *Secretary.*

### CONTENTS.

### DENTAL REGISTER,

### OF THE WEST.

Issued Monthly, at $3 a year in advance.

DEVOTED TO THE INTERESTS OF THE DENTAL PROFESSION.

**Edited by J. TAFT and GEO. WATT.**

The Volume commences in January and closes in December.

The Register being issued monthly is always fresh, therefore indispensable to the practitioner who desires to keep posted up with the new inventions and improvements which are daily developed. Neither expense nor effort will be spared to give value and variety to its contents. Articles will be illustrated with well executed engravings, whenever they will add to the interest or assist in explanation.

Its extensive circulation and frequency of issue makes it the BEST DENTAL ADVERTISING MEDIUM in the United States.

☞CONTRIBUTIONS SOLICITED.

Address Original Communications to **Geo. Watt, Xenia, O.**

Exchanges to **J. Taft**, or **Dental Register, Cincinnati, Ohio.**

Business Communications to

J. TAFT, Publisher,

172 Race Street, Cincinnati, O.

JNO. T. TOLAND, Proprietor,

38 West Fourth Street, Cincinnati, O.

Communications must reach the Editor as early as the 15th, to insure insertion in the next issue.

